# PROMOTING GERONTOLOGICAL TRAINING AND POSITIVE ATTITUDES OF AGING THROUGH HIGH-IMPACT TEACHING PRACTICES

**DOI:** 10.1093/geroni/igae098.3564

**Published:** 2024-12-31

**Authors:** Aimee Fox, Bethany Riding

**Affiliations:** Utah Valley University, Orem, Utah, United States; Utah Valley University, Orem, Utah, United States

## Abstract

Despite being the youngest state, Utah, along with the rest of the United States, has a growing aging population. There is demand from state leaders and employers for individuals, families, and a workforce that are knowledgeable about the experiences and needs of older adults. Utah Valley University (UVU) is Utah’s largest public university, with a dual-mission of a first-rate teaching university and open-admissions promoting easy access to higher education. Yet, efforts at UVU to promote gerontological training in the social sciences have been minimal. Students at UVU are often unaware of what gerontology is and the importance of gerontological training, and report negative attitudes and fears about aging and older adults. This descriptive case study is the first stage of a larger project aimed at promoting enrollment in UVU’s gerontology certificate and shifting student attitudes about aging to promote positive perceptions of and interactions with older adults and aging experiences. During this first stage, the gerontology certificate was adjusted to align with AGHE gerontology competencies, and an Adult Development and Aging course was re-designed with high-impact practices, including a common intellectual experience focused on service- and experiential-learning activities. This study reports on re-design efforts, the engagement and feedback of 80 students across two sections of the course, and the impact of learning activities on students’ attitudes about aging and older adults. Also addressed are theoretical and practical suggestions for promoting gerontological training, potential pitfalls to avoid, and recommendations for implementing these changes across a variety of higher education settings.

